# A seven-year surveillance study of the epidemiology, antifungal susceptibility, risk factors and mortality of candidaemia among paediatric and adult inpatients in a tertiary teaching hospital in China

**DOI:** 10.1186/s13756-020-00798-3

**Published:** 2020-08-14

**Authors:** Zhangrui Zeng, Yinhuan Ding, Gang Tian, Kui Yang, Jian Deng, Guangrong Li, Jinbo Liu

**Affiliations:** Department of Laboratory Medicine, the Affiliated Hospital of Southwest Medical University, 25 Taiping street, Luzhou, 646000 P.R. China

**Keywords:** Candidaemia, Epidemiology, Paediatric patients, Adult patients, Risk factors

## Abstract

**Background:**

There are no current national estimates of the candidaemia burden in China, and epidemiological candidaemia data from the underdeveloped region of China are lacking.

**Methods:**

A 7-year retrospective study was carried out to analyse the prevalence, species distribution, antifungal susceptibility, risk factors and inpatient mortality of candidaemia among paediatric and adult patients in a regional tertiary teaching hospital in China.

**Results:**

During the seven-year study period, a total of 201 inpatients with candidaemia were identified. The median age of the patients was 65 years (range, 1 day to 92 years), and 114 of the patients (56.7%) were male. The mean annual incidence of candidaemia was 0.26 cases per 1000 admissions (0.42 cases per 1000 paediatric admissions vs 0.24 cases per 1000 adult admissions, *P* < 0.05). *Candida albicans* was the most common fungal species (81/201, 40.3%) in all patients, *Candida glabrata* was the most common fungal species (18/35, 51.4%) in paediatric patients. Most isolates were susceptible to flucytosine (99.0%) and amphotericin B (99.0%), and the activity of antifungal agents against Candida species was no significant difference in satisfaction between paediatric and adult patients (*P* > 0.05). The all-cause mortality rate was 20.4% (paediatric patients: 11.4% vs adult patients:22.3%, *P* > 0.05). Fewer univariate predictors of poor outcomes were identified for paediatric patients than for adult patients (4 vs 11 predictors). Respiratory dysfunction and septic shock were independent predictors of 30-day mortality for all patients.

**Conclusions:**

The epidemiological data of candidaemia in paediatric and adult patients are only different in the distributions of *Candida* species and the mean annual incidence of candidaemia. Flucytosine and amphotericin B can be used as first-choice agents when no antifungal susceptibility test results are available.

## Background

Candidaemia is the most common fungal disease among hospitalised patients worldwide and is the fourth to tenth most common bloodstream infection (BSI) in most population-based studies [1, 2]. Candidaemia is associated with significant morbidity and mortality [3]. The main risk factors for candidaemia include critical illness, a long intensive care unit (ICU) length of stay, haematologic malignant disease, solid-organ transplantation, solid-organ tumours, low birth weight in neonates and preterm infants, broad-spectrum antimicrobial agent use, central venous catheterization (CVC), total parenteral nutrition, haemodialysis, abdominal surgery, and aggressive chemotherapy [1]. With the increase in related research, reports have shown that the incidence of candidaemia is age-specific, with maximum rates observed in those with older age (over 65 years) [1, 4, 5].

More than 40 Candida species can cause candidaemia in humans [6]. Five species of Candida (*Candida albicans*, *Candida glabrata*, *Candida parapsilosis*, *Candida tropicalis* and *Candida krusei*) are the most common species and account for more than 90% of all the isolates [2]. The variability in the relative proportions of Candida isolates has been associated with clinical condition or risk factors such as age, underlying comorbidities, the extensive use of antifungal agents and geography. *Candida albicans* is the primary cause of candidaemia and one of the most common species in many countries, *Candida glabrata* is the second or third most common species in the USA and Europe, and *Candida parapsilosis* is predominant in neonates in South America, southern Europe and Asia [2]. The global incidence of candidaemia varies from 0.3 to 5 per 1000 admissions according to geographical region, local epidemiology, age and other factors [7]; the 30-day mortality among all patients with candidaemia has been reported to be between 22 and 70% [8], and the cost of candidaemia treatment has been reported to be US $40,000 per patient [1, 9, 10].

In China, the epidemiology of candidaemia varies widely among different areas [11]. Epidemiological surveillance of candidaemia has focused on ICUs and single centres in China, and national surveillance systems are usually absent. Most of the existing epidemiological surveillance of candidaemia has focused on adults or children, and little information about general populations (including neonates, children and adults) is known. Therefore, in the present study, we performed a seven-year retrospective study to evaluate the epidemiology, antifungal susceptibility, risk factors and mortality of candidaemia among all inpatients in a tertiary teaching hospital in China.

## Methods

### Patient data collection

We conducted a retrospective observational study of electronic laboratory records. The fungal specimen data were collected from inpatients with candidaemia in the Affiliated Hospital of Southwest Medical University (Luzhou, China), which is a 3200-bed tertiary care teaching hospital with 43 wards and approximately 120,000 annual admissions, from January 2013 to December 2019. The diagnostic criteria of candidaemia were based on the guidelines for the diagnosis and treatment of Candidiasis: the expert consensus issued by the Chinese Medical Association [12]; these criteria were also in accordance with the European Society of Clinical Microbiology and Infectious Diseases (ESCMID)* guidelines for the diagnosis and management of *Candida* diseases 2012 [13, 14] and the Infectious Diseases Society of America (IDSA) Guidelines for the Management of Candidiasis: 2016 Update [15]. For each patient, only the first episode was included in our analysis. Patient cultures with two or more *Candida* species were excluded from the analysis, and all data were collected from electronic medical records. The following data were retrospectively collected from all patients: demographic characteristics, underlying comorbidities, *Candida* species, susceptibility to antifungal agents and mortality. Data on the following risk factors associated with candidaemia were also collected: gestational age and weight of neonates, indwelling central vascular catheter, mechanical ventilation, systemic corticosteroid treatment (a dose equivalent to prednisone 10 mg/d for at least 14 days), total parenteral nutrition, chemotherapy, abdominal surgery, ICU admission, neutropenia (absolute neutrophil count < 500 cells/μl), concomitant bacterial infections, septic shock, haemodialysis, broad-spectrum antibiotic use and treatment with antifungal agents. The study protocol was approved by the ethics committee of the hospital (Project No. KY2020043). The need for informed consent was waived by the Clinical Research Ethics Committee.

### Microorganism identification and antifungal susceptibility

According to the manufacturer’s instructions, blood was inoculated into both aerobic and anaerobic BacT/AlerT 3D vials (Bruker Diagnostics Inc., USA). All positive cultures were manually sampled and inoculated onto CHROMagar *Candida* medium (CHROMagar Company, France) to ensure viability and purity. The identification of all species was confirmed by a MicroScan WalkAway 96 Plus System (Siemens, Germany) and Microflex LT (Bruker Diagnostics Inc., USA) matrix-assisted laser-desorption/ionization time-of-flight (MALDI-TOF) mass spectrometry (MS) system.

Antifungal susceptibility tests for fluconazole (FCA), itraconazole (ITR), voriconazole (VRC), flucytosine (5-FC) and amphotericin B (AMB) were performed for all *Candida* strain isolates by using an ATB FUNGUS 3 kit (bioMérieux, France). The minimal inhibitory concentrations (MICs) of the antifungal agents were judged by visualization in our laboratory according to the manufacturer’s instructions. The quality control strains were *C. parapsilosis* ATCC 22019 and *C. krusei* ATCC 6258. The results were interpreted using the Clinical and Laboratory Standards Institute M27-A3 microbroth dilution method.

### Statistical analyses

The data were analysed using Microsoft Excel (version 2016, Redmond, USA) and IBM SPSS software version 24 for Windows (IBM, Armonk, USA). Categorical data were compared using chi-square or Fisher’s exact tests. Continuous data were analysed using Student’s t-test or the Mann-Whitney U test. Multivariable logistic regression analysis was performed to identify independent predictors of candidemia and 30-day hospital mortality. Biologically plausible variables with a value of *P* < 0.1 according to the univariate analyses were included in the multiple logistic regression model. Statistical significance was determined using two-tailed tests, and *P* < 0.05 was considered statistically significant.

## Results

A total of 201 distinct candidaemia episodes were identified during our study period. The median age was 65 years (range 1 day − 92 years), and 114 patients (56.7%) were male. Most candidaemia episodes were diagnosed in medical wards (89, 44.3%), followed by ICUs (46, 22.9%), paediatric wards (35, 17.4%) and surgical wards (31, 15.4%). Most of the patients had one or more comorbidities. Pulmonary infection (49.8%), chronic/acute renal failure (45.3%) and cardiovascular disease (42.8%) were the most common underlying comorbidities, followed by neurological diseases (38.8%), diabetes mellitus (29.9%), respiratory dysfunction (28.9%), gastrointestinal pathologies (28.9%) and chronic/acute liver disease (24.4%). Moreover, the most common underlying conditions documented prior to candidaemia were prior exposure to broad-spectrum antibiotics (89.1%), treatment with antifungal agents (56.7%), concomitant bacterial infections (54.7), total parenteral nutrition (47.3%), mechanical ventilation (43.3%), ICU/paediatric ICU (PICU)/neonatal ICU (NICU) admission (40.3%) and CVC (38.3%). In total, 53 (26.4%, 53/201) patients had received previous antifungal treatment, and paediatric patients accounted for 71.4% (25/35) of the total. The underlying comorbidities in adult patients were significantly worse than those in paediatric patients, but the number of underlying conditions in paediatric patients were significantly higher than those in adult patients, and the difference was statistically significant (*P* < 0.05). FCA was the most frequently used empirical antifungal treatment (60/114, 52.6%). The demographic and clinical characteristics of the patients are summarized in Table 1 and Table 2.

**Table 1:** Distribution and incidence of Candida species

	*Candida* species						
	Total	*C. albicans*	*C. glabrata*	*C. tropicalis*	*C. parapsilosis*	*C. krusei*	*others*
	(*n* = 201) 100.0%	(*n* = 81) 40.3%	(*n* = 73) 36.3%	(*n* = 28) 13.9%	(*n* = 8) 4.0%	(*n* = 6) 3.0%	(*n* = 5) 2.5%
Distribution n(%)							
Paediatric patients (≤16 years)	35(17.4)	16(45.7)	18 (51.4)	0 (0)	0 (0)	0 (0)	1 (2.9)
0–28 days	32(15.9)	16(50.0)	15(46.9)	0 (0)	0 (0)	0 (0)	1 (3.1)
29 days - 1 year	1(0.5)	0 (0)	1(100.0)	0 (0)	0 (0)	0 (0)	0 (0)
2–16 years	2(1.0)	0 (0)	2(100.0)	0 (0)	0 (0)	0 (0)	0 (0)
Adult patients (> 16 years)	166(82.6)	65(39.2)	55(33.1)	28(16.9)	8(4.8)	6(3.6)	4(2.4)
17–49 years	52 (25.9)	21(40.4)	19 (36.5)	7(13.5)	1(1.9)	3(5.8)	1 (1.9)
50–65 years	56 (27.9)	11 (19.6)	23 (41.1)	14 (25.0)	5(8.9)	1(1.8)	2 (3.6)
> 65 years	58(28.8)	33(56.9)	13(22.4)	7(12.1)	2(3.4)	2(3.4)	1(1.7)
Gender							
Male	114 (56.7)	44 (38.6)	39 (34.2)	19 (16.7)	6(5.3)	2 (1.7)	4 (3.5)
Female	87 (43.3)	37 (42.5)	34 (39.1)	9(10.3)	2(2.3)	4 (4.6)	1 (1.1)
Incidence (episodes/1000 admissions)							
2013	0.20	0.06	0.12	0.01	0.00	0.01	0.00
2014	0.22	0.09	0.10	0.03	0.00	0.00	0.01
2015	0.27	0.10	0.12	0.02	0.00	0.03	0.00
2016	0.37	0.23	0.10	0.04	0.00	0.01	0.00
2017	0.32	0.10	0.14	0.07	0.01	0.00	0.00
2018	0.16	0.06	0.07	0.02	0.00	0.00	0.00
2019	0.26	0.08	0.04	0.05	0.05	0.01	0.03
Mean annual incidence	0.26	0.10	0.09	0.04	0.01	0.01	0.01

*Others include *C. guilliermondii* (3)*, C. haemulonii* (3) and *C. inconspicua* (1)

**Table 2:** Patient characteristics and incidence (episode/1000 admission)

	All patients	Child patients(0-16 years)	Adult patients(> 16 years)	P*
	(*n* = 201) 100.0%	(*n* = 35) 17.4%	(*n* = 166) 82.6%	
**Age (median, range)**	**65 years** **(1 day, 92 years)**	**1 day** **(1 day,5 years)**	**61 years** **(18 years, 92 years)**	**< 0.001**
Gender (male:female)	114:87	22:13	92:74	0.420
Length of hospital stay(days)	36.9 ± 39.5	41.5 ± 20.9	30.6 ± 39.6	0.117
Underlying comorbidities (*n*, %)				
Gastrointestinal perforation	24 (11.9)	2 (5.7)	22(13.3)	0.211
***Respiratory dysfunction***^***a***^	***58 (28.9)***	***3 (8.6)***	***55 (33.1)***	***0.004***
**Pulmonary infection**	**100(49.8)**	**24 (68.6)**	**76(45.8)**	**0.014**
***Cardiovascular disease***	***86 (42.8)***	***3(8.6)***	***83 (50.0)***	***< 0.001***
**Neurological diseases**	**78 (38.8)**	**24(68.6)**	**54 (32.5)**	**< 0.001**
***Gastrointestinal pathology***^***b***^	***58(28.9)***	***2(5.7)***	***43 (25.9)***	***0.001***
Chronic/acute liver disease	49(24.4)	9(25.7)	40(24.1)	0.839
***Chronic/acute renal failure***^***c***^	***91(45.3)***	***9(25.7)***	***82 (49.4)***	***0.011***
Solid tumour	15(7.5)	0(0)	15(9.0)	0.065
Haematological malignancy	11 (5.5)	2 (5.7)	9(5.4)	0.697
**Congenital malformations/syndromes**	**6(3.0)**	**3 (8.6)**	**3 (1.8)**	**< 0.001**
***Diabetes mellitus***	***60 (29.9)***	***0(0)***	***60 (36.1)***	***< 0.001***
**Hematologic (nonmalignant)**	**29 (14.4)**	**10 (28.6)**	**19 (11.4)**	**0.009**
HIV/AIDS	10 (5.0)	0(0)	10 (6.0)	0.136
Severe trauma	17(8.5)	2 (5.7)	15 (9.0)	0.521
Risk factors (*n*, %)				
***Presence of CVC***^***d***^	***77(38.3)***	***7(20.0)***	***70(42.2)***	***0.014***
***Other invasive catheters***	***60(29.9)***	***5(14.3)***	***55(33.1)***	***0.027***
**Mechanical ventilation**	**87(43.3)**	**21 (60.0)**	**66(39.8)**	**0.028**
Receipt of corticosteroids^e^	42 (20.9)	9(25.7)	33(19.9)	0.440
Total parenteral nutrition	95(47.3)	18(51.4)	77(46.4)	0.587
Malnutrition	55(27.4)	9(25.7)	46(27.7)	0.810
Chemotherapy	20(10.0)	2 (5.7)	18(10.8)	0.357
**Hemodialysis**	**30(16.9)**	**0(0)**	**30(18.1)**	**0.006**
***Abdominal surgery***^***f***^	***31 (15.4)***	***0 (0)***	***31 (18.7)***	***0.005***
**ICU/PICU/NICU**	**81(40.3)**	**35 (100.0)**	**46 (27.7)**	**< 0.001**
Neutropenia^g^	16(8.0)	0 (0)	16(9.6)	0.056
**Concomitant bacterial infections**	**110(54.7)**	**30 (85.7)**	**80 (48.2)**	**< 0.001**
Septic shock	39 (19.4)	3 (8.6)	36(21.7)	0.075
**Broad-spectrum antibiotics**	**179 (89.1)**	**35 (100.0)**	**144 (86.7)**	**0.022**
**Treatment with antifungal agents**	**114 (56.7)**	**27(77.1)**	**87(52.4)**	**0.007**
*C. albicans*	81(40.3)	16(45.7)	65(39.2)	0.472
***C. glabrata***	**73(36.3)**	**18(51.4)**	**55(33.1)**	**0.041**
***C. tropicalis***	***28(13.9)***	***0(0)***	***28(16.9)***	***0.009***
Death	41(20.4)	4(11.4)	37(22.3)	0.113
Incidence(n,episodes/1000 admissions)				
2013	14(0.20)	0(0.0)	14(0.24)	0.248
2014	23(0.22)	1(0.09)	22(0.24)	0.507
2015	29(0.27)	6(0.53)	23(0.24)	0.117
**2016**	**42(0.37)**	**12(1.07)**	**30(0.29)**	**0.001**
**2017**	**38(0.32)**	**12(1.06)**	**26(0.24)**	**< 0.001**
2018	20(0.16)	3(0.21)	17(0.15)	0.480
2019	35(0.26)	1(0.08)	34(0.27)	0.252
Mean annual incidence	201(0.26)	35(0.42)	166(0.24)	0.002

*Statistical results of demographic characteristics of pediatric and adult patients

^a^ Includes the following diseases: chronic obstructive pulmonary disease and acute respiratory distress syndrome

^b^ Includes the following diseases: cholecystitis, pancreatitis, and peritonitis

^c^ Chronic/Acute renal failure is the permanent or sudden and often temporary loss of kidney function with N waste retention and hypourocrinia

^d^ *CVC* central venous catheter

^e^a dose equivalent to the prednisone dosage of 0.3 mg/kg/day for at least 14 days

^f^ including: gastrointestinal perforations, severe acute pancreatitis and complex ventral hernia

^g^ Neutropenia is the absolute neutrophil count, that is, < 500 cells/μl

The mean annual incidence of candidaemia was 0.26/1000 admissions, including 0.42/1000 paediatric admissions (1.61/1000 neonatal admissions (age < 28 days), 0.06/1000 infant admissions (28 days < age < 1 year) and 0.04/1000 child admissions (1 year < age < 16 years)) and 0.24/1000 adult admissions (0.09/1000 surgical admissions, 0.30/1000 medical admissions and 1.64/1000 ICU admissions). According to the Candida species, the incidence of the three most commonly isolated Candida species were as follows: *C. albicans,* 0.10/1000 admissions; *C. glabrata,* 0.09/1000 admissions; and *C. tropicalis,* 0.04/1000 admissions.

**Fig. 1:**
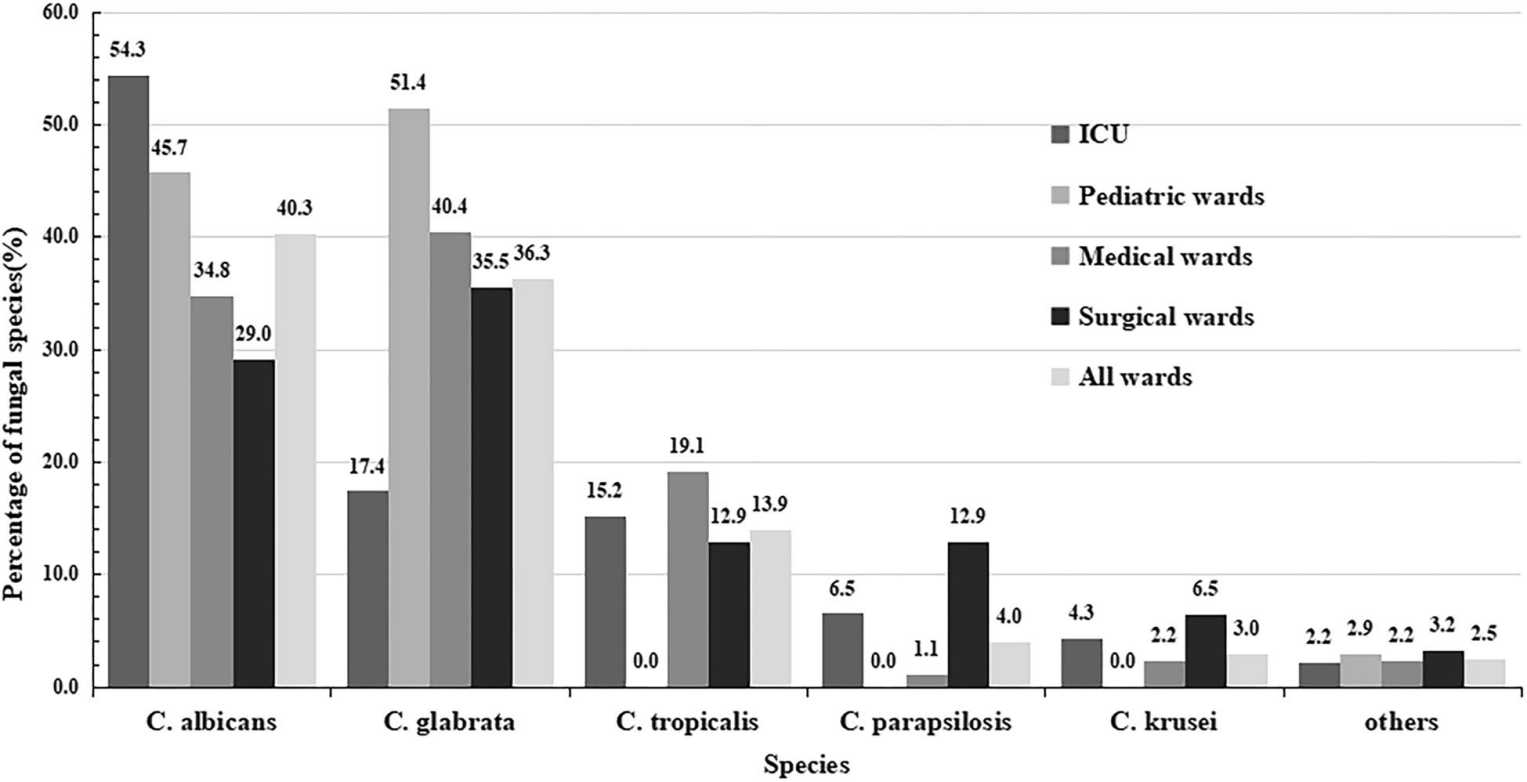
Distribution of the fungal species according to different wards. FootNote: Others include *C. guilliermondii* (3), C. haemulonii (1) and *C. inconspicua* (1)

The most common species among all Candida species isolates was *C. albicans* (40.3%), followed by *C. glabrata* (36.3%), *C. tropicalis* (13.9%), *C. parapsilosis* (4.0%), *C. krusei* (3.0%) and others (2.5%). The distributions of Candida species in paediatric (< 16 years) and adult (≥16 years) patients are shown in Table 1. In patients aged 0–16 years and 49–65 years, *C. glabrata* was the predominant species (51.4 and 41.1%, respectively), but in patients aged 17–49 and > 65 years, *C. albicans* was the main species (45.7 and 56.9%, respectively). The distribution of Candida species in paediatric, surgical, internal medicine and ICU wards is shown in Fig. 1.

The results of in vitro susceptibility testing of *Candida* strain isolates are summarized in Table 3. All isolates were highly susceptible to AMB (99.0%) and 5-FC (99.0%). The resistance rates of ITR, VRC and FCA were 24.9 19.4 and 18.5%, respectively. *C.tropicalis* had the highest antifungal agent resistance rate among the *Candida* species and was resistant to FCA (39.3%), ITR (39.3%) and VRC (42.9%). The activity of antifungal agents against Candida species was not significantly different in terms of satisfactory outcomes between paediatric and adult patients (*P* > 0.05). The detailed data are shown in Table 3.

**Table 3:** In vitro antifungal susceptibility testing of 201 clinical isolates into 5 antifungal agents

Species (No of isolates)	Antifungal agent	Resistant n(%)^e^			
		Children (35)	Adults(166)	total	P^c^
*Candida albicans*(81)	Amphotericin B	0	0	0^b^	–
	Flucytosine	0	1(1.5)	1(1.2)^b^	0.618
	Fluconazole	2(12.5)	17(26.2)	19(23.5)	0.248
	Itraconazole	8(50.0)	22(33.8)	30(37.0)^b^	0.231
	Voriconazole	4(25.0)	20(30.8)	24(29.6)^b^	0.651
*Candida glabrata*(73)	Amphotericin B	1(5.6)	0	1(1.4)^b^	0.078
	Flucytosine	0	0	0^b^	–
	Fluconazole	1(5.6)	5(9.1)	6(8.2)	0.635
	Itraconazole	2(11.1)	5(9.1)	7(9.6)^b^	0.801
	Voriconazole	1(5.6)	2(3.6)	3(4.1)^b^	0.722
*C.tropicalis*(28)	Amphotericin B	0	1(3.6)	1(3.6)^b^	–
	Flucytosine	0	1(3.6)	1(3.6)^b^	–
	Fluconazole	0	11(39.3)	11(39.3)^b^	–
	Itraconazole	0	11(39.3)	11(39.3)^b^	–
	Voriconazole	0	12(42.9)	12(42.9)^b^	–
*C. krusei*(6)	Amphotericin B	0	0	0	–
	Flucytosine	0	0	0	–
	Fluconazole^a^	–	–	–	–
	Itraconazole	0	1(16.7)	1(16.7)^b^	–
	Voriconazole	0	1(16.7)	1(16.7)^b^	–
All of isolates (201)	Amphotericin B	1(2.9)	1(0.6)	2(1.0)	0.222
	Flucytosine	0(0)	2(1.2)	2(1..0)	0.513
	Fluconazole^a^	3(8.6)	33(20.6)	36(18.5)	0.096
	Itraconazole	10(28.6)	40(24.1)	50(24.9)	0.578
	Voriconazole	5(14.3)	34(20.5)	39(19.4)	0.400

*MIC* minimal inhibitory concentration

^a^Resistance rate was based on the intrinsic resistance of *C*. *krusei* and did not follow the actual MICs

^b^The breakpoints of *Candida* spp. according to the manufacturer’s instructions of ATB FUNGUS 3 system

^c^The difference of resistance rate between children and adults was analyzed by chi square test

^e^*C. parapsilosis* and others isolates (*C. guilliermondii* (3), *C. haemulonii* (1) and *C. inconspicua* (1)) were susceptible to five antifungal agents (100%), so the results were not listed

The all-cause mortality rate in the 201 patients was 20.4% (41/201). The 7-day and 30-day mortality rates were 8.5% (17/201) and 17.9% (36/201), respectively. The mortality rates of *C. albicans*, *C. glabrata*, *C. tropicalis* and *C. parapsilosis* infections were 27.2% (22/81), 16.4% (12/73), 21.4% (6/28) and 12.5% (1/8), respectively. The mortality rates for paediatric wards, medical wards, surgical wards and ICU wards were 11.4% (4/35), 22.5% (20/89), 16.1% (5/31) and 26.1% (12/46), respectively. The mortality rates for different age groups were 11.4% (4/35, 0–16 years) among paediatric patients and 22.3% (37/166(> 16 years), 7.7% (4/52, 17–49 years), 19.6% (11/56, 50–65 years) and 37.9% (22/58, > 65 years)) among adult patients.

The univariate predictors of poor outcomes due to candidaemia are shown in Table 4. For paediatric patients with candidaemia, the variables associated with 30-day mortality were as follows: length of hospital stay, respiratory dysfunction, chronic/acute renal failure and septic shock. For adult patients with candidaemia, the variables associated with 30-day mortality were as follows: age, length of hospital stay, respiratory dysfunction, pulmonary infection, cardiovascular disease, chronic/acute renal failure, other invasive catheters, mechanical ventilation, septic shock, *C. albicans* infection, concomitant bacterial infection and haematologic (nonmalignant) disease. The results of the multivariate analysis are listed in Table 5. Because the total numbers of paediatric patients (35 patients) and deaths (3 patients) were very small, multivariable logistic regression analysis was not performed for paediatric patients. Respiratory dysfunction and septic shock were independent predictors of 30-day mortality in all patients and adult patients. The length of hospital stay was a protective factor for 30-day mortality in all patients and adult patients, and other invasive catheters were only the protective factor for 30-day mortality in all patients. The prognostic factors for 30-day mortality in all patients and adult patients were almost the same, and the independent predictors were the same, with no significant differences (Table 5). In addition, we compared the independent protective factors and independent risk factors in this study with those in other studies and found that the protective factors (length of hospital stay and other invasive catheters) in our study were different from those in other studies, while the independent risk factor of septic shock in our study was also identified in some others studies, but the independent risk factor of respiratory dysfunction was not reported in other studies (Table 6).

**Table 4:** Factors associated with 30-days mortality by univariate analysis in inpatients with candidaemia

Variable	Adult patients (> 16 years) 30-days outcome		*P*-value	Child patients (0–16 years) 30-days outcome		*P*-value	All patients 30-days outcome		*P*-value
	Survived (*n* = 133)	Died (*n* = 33)		Survived (*n* = 32)	Died (*n* = 3)		Survived (*n* = 165)	Died (*n* = 36)	
**Median age(range)**	**60 years** **(18, 92 years)**	**67 years** **(29, 86 years)**	**0.001**	1 days (1 day, 5 year)	1 days (1 day,1 day)	0.585	**52 years** **(1 day, 92 years)**	**65 years** **(1 day, 86 years)**	**0.001**
Gender (male:female)	72:61	20:13	0.503	19:13	3:0	0.164	91:74	23:13	0.557
**Length of hospital stay(days)**	**35.3 ± 42.8**	**11.7 ± 9.2**	**0.002**	**43.8 ± 20.2**	**16.7 ± 8.5**	**0.029**	**36.9 ± 39.5**	**12.1 ± 9.1**	**< 0.001**
Underlying comorbidities (*n*, %)									
Gastrointestinal perforation	15(11.3)	7(21.2)	0.132	2(6.3)	0(0)	0.656	17 (10.3)	7 (19.4)	0.125
**Respiratory dysfunction**	**28(21.1)**	**27(81.8)**	**< 0.001**	**1(3.1)**	**2(66.7)**	**< 0.001**	**29(17.6)**	**29 (80.6)**	**< 0.001**
**Pulmonary infection**	**54(40.6)**	**22(66.7)**	**0.007**	22(68.8)	2(66.7)	0.941	**76(46.1)**	**24(66.7)**	**0.025**
**Cardiovascular disease**	**56(42.1)**	**27(81.8)**	**< 0.001**	3(9.4)	0(0)	0.579	**59 (35.8)**	**27 (75.0)**	**< 0.001**
Neurological diseases	41(30.8)	13(39.4)	0.347	22(68.8)	2(66.7)	0.941	63 (38.2)	15 (41.7)	0.697
Gastrointestinal pathology	42(31.6)	14(42.4)	0.238	2(6.3)	0(0)	0.656	44(26.7)	14 (38.9)	0.143
Chronic/acute liver disease	30(22.6)	10(30.3)	0.352	9(28.1)	0(0)	0.287	39 (23.6)	10 (27.8)	0.600
**Chronic/acute renal failure**	**60(45.1)**	**22(66.7)**	**0.027**	**6(18.8)**	**3(100.0)**	**0.002**	**66 (40.0)**	**22 (61.1)**	**0.001**
Haematological malignancy	4(3.0)	3(9.1)	0.120	2(6.3)	2()	0.656	6(36.4)	5(13.9)	0.217
Solid tumour	12(9.0)	3(9.1)	0.990	0(0)	0(0)	–	12(7.3)	3 (8.3)	0.826
Severe autoimmune diseases	12(9.0)	3(9.1)	0.990	0(0)	0(0)	–	12(7.3)	3 (8.3)	0.826
Congenital malformations/syndromes	0	0	–	5(15.6)	1(33.3)	0.365	5 (3.0)	1 (2.8)	0.799
***Hematologic (nonmalignant)***	**12(9.0)**	**7(21.2)**	**0.049**	9(28.1)	1(33.3)	0.849	21 (12.7)	8 (22.2)	0.142
Diabetes mellitus	45(33.8)	15(45.5)	0.214	0(0)	0(0)	–	45(27.3)	15 (41.7)	0.087
HIV/AIDS	9(6.8)	1(3.0)	0.419	0(0)	0(0)	–	9(5.5)	1 (2.8)	0.503
Severe trauma	12(9.0)	3(9.1)	0.990	2(6.3)	0(0)	0.565	14(8.5)	3 (8.3)	0.976
Risk factors (*n*, %)									
premature neonates≤36 weeks)^a^	–	–	–	28(87.5)	2(66.7)	0.515	28(93.3)	2(100.0)	0.706
Very low birth weight neonates(<1500 g)^a^	–	–	–	19(59.4)	2(66.7)	0.886	19(63.3)	2(100.0)	0.290
Presence of CVC	57(42.9)	13(39.4)	0.718	6(18.8)	1(33.3)	0.546	63(38.2)	14 (38.9)	0.937
**Other invasive catheters**	**51(38.3)**	**4(12.1)**	**0.004**	5(15.6)	0(0)	0.460	**56(33.9)**	**4(11.1)**	**0.007**
**Mechanical ventilation**	**45(33.8)**	**21(63.6)**	**0.002**	19(59.4)	2(66.7)	0.805	**64(38.8)**	**23 (63.9)**	**0.006**
Receipt of corticosteroids	27(20.3)	6(18.2)	0.785	9(18.1)	0(0)	0.287	36 (21.8)	6(16.7)	0.491
Total parenteral nutrition	57(42.9)	20(60.6)	0.067	17(53.1)	1(33.3)	0.512	74(44.8)	21(58.3)	0.142
Malnutrition	36(27.1)	10(30.3)	0.710	8(25.0)	1(33.3)	0.752	44(26.7)	11(30.6)	0.635
Chemotherapy	16(12.0)	2(6.1)	0.324	2(6.3)	0(0)	0.656	18 (10.9)	2 (5.6)	0.331
Abdominal surgery	24(18.0)	7(21.2)	0.676	0(0)	0(0)	–	24(14.5)	7 (19.4)	0.461
Hemodialysis	22(16.5)	8(24.2)	0.303	0(0)	0(0)	–	22(13.3)	8(22.2)	0.175
ICU/PICU/NICU	33(24.8)	13(39.4)	0.094	32(100.0)	3(100.0)	–	65(39.4)	16 (44.4)	0.576
Neutropenia^g^	13(9.8)	3(9.1)	0.905	0(0)	0(0)	–	13(7.9)	3 (8.3)	0.927
***Concomitant bacterial infections***	**59(44.4)**	**21(63.6)**	**0.047**	27(84.4)	3(100.0)	0.460	86 (52.1)	24 (66.7)	0.112
**Septic shock**	**8(6.0)**	**28(84.8)**	**< 0.001**	**1(3.1)**	**2(66.7)**	**< 0.001**	**9 (5.5)**	**30 (83.3)**	**< 0.001**
Broad-spectrum antibiotics	115(86.5)	29(87.9)	0.830	32(100.0)	3(100.0)	–	147(89.1)	32(88.9)	0.972
Treatment with antifungal agents	71(53.4)	16(48.5)	0.614	24(75.0)	3(100.0)	0.324	95(57.6)	19(52.8)	0.599
Species, *n* (%)									
***C. albicans***	**47(35.3)**	**18(54.5)**	**0.043**	16(50.0)	0(0)	0.096	63 (38.2)	18 (50.0)	0.190
*C*. *glabrata*	46(34.6)	9(27.3)	0.424	15(46.9)	3(100.0)	0.078	61 (37.0)	12 (33.3)	0.681

*ICU* intensive care unit; *PICU* pediatric intensive care unit, *NICU* neonatal intensive care unit, *CVC* central venous catheter

^a^Only neonatal cases were analyzed

**Table 5:** Factors associated with 30-days mortality by multivariate analysis^a^

Variable	All patients			Adult patients		
	Odds ratio	95% confidence interval	*P*-value	Odds ratio	95% confidence interval	*P*-value
Median age	1.02	0.973–1.065	0.444	1.03	0.957–1.109	0.427
**Length of hospital stay(days)**	**0.88**	**0.809–0.964**	**0.005**	**0.89**	**0.802–0.99**	**0.032**
**Respiratory dysfunction**	**13.78**	**2.254–84.198**	**0.005**	**22.57**	**2.014–252.84**	**0.011**
Pulmonary infection	0.68	0.125–3.693	0.655	0.98	0.142–6.743	0.982
Cardiovascular disease	0.65	0.088–4.787	0.672	3.36	0.269–41.933	0.347
Chronic/acute renal failure	2.50	0.464–13.425	0.287	1.19	0.191–7.392	0.854
**Other invasive catheters**	**0.04**	**0.002–0.695**	**0.028**	0.04	0.001–1.233	0.066
Mechanical ventilation	4.59	0.554–37.999	0.158	12.56	0.981–160.793	0.052
**Septic shock**	**99.97**	**11.997–832.995**	**< 0.001**	**89.72**	**10.161–792.184**	**< 0.001**
Diabetes mellitus^b^	0.12	0.013–1.038	0.054	–	–	–
C. Albicans^b^	–	–	–	3.16	0.391–25.505	0.281
Concomitant bacterial infection^b^	–	–	–	4.97	0.42–58.742	0.204
Hematologic (nonmalignant)^b^	–	–	–	0.27	0.018–4.057	0.346
Total parenteral nutrition^b^	–	–	–	0.06	0.004–1.06	0.055
ICU/PICU/NICU^b^	–	–	–	0.31	0.029–3.259	0.328

*ICU* intensive care unit; *PICU* pediatric intensive care unit, *NICU* neonatal intensive care unit

^a^Because the total number of pediatric patients(35 patients) and deaths (3 patients) were very small, multivariable logistic regression analysis was not performed in pediatric patients

^b^ Biologically plausible variables with a value of *P* > 0.1 according to the univariate analyses were not included in the multiple logistic regression model

**Table 6:** Protective factor and predictors of 30-day mortality in others studies

Authors	Country or region	study period	study design	samples	No of samples	Protective factor	Predictors of 30-day mortality	Reference
Ma et al	China	2009–2011	Retrospective, observational, single-center study	Candidemia((130 adults and 3 children < 15 years patients)	133		Presence of CVC	37
Cortes et al	Colombia	2008–2009	Retrospective, observational, multicenter study (seven tertiary-care hospitals)	Candidemia(9 days to 87 years patients)	131	Fluconazole therapy	Age, the presence of shock at the time of Candida detection	40
Wang et al	China	2008–2010	Retrospective, multicentre study (4 tertiary general hospitals)	Candidemia(> 16 years patients)	147	Antifungal therapy administered before microbiological documentation	Absence of antifungal therapies, receipt of mechanical ventilation and APACHE II score ≥ 20	41
Tedeschi et al	Italy	2012–2013	Retrospective, observational, multicenter cohort study(39 hospitals)	Candidemia (adult patients)	232	Central-venous-catheter removal and adequate and timely(within 72 h of drawing blood cultures) therapy	Chronic-obstructive-pulmonary-disease and isolation of C. tropicalis	42
Li et al	China	2010–2014	Retrospective, observational, single-center study	Candidemia(> 18 years patients)	190	Proven catheter-related candidemia	Severe sepsis or septic shock	35
Gonzalez-Lara et al	Mexico	2008–2014	Retrospective, laboratory-based survey study(two tertiary-care centers hospitals)	Candidemia(all patients)	149	Early CVC withdrawal andempirical antifungal therapy	Severe sepsis and previous diagnosis of cirrhosis	43
Jia et al	China	2011–2016	Retrospective, observational, multicenter study (3 hospitals)	Candidemia(15–90 years patients)	198		ICU admission, catheter-relatedcandidemia, ascites, septic shock and concomitant bacterial infection	18
Ortega-Loubon et al	Spain	2007–2016	Retrospective, observational, single-cente study	Candidemia(> 18 years patients)	296		Prolonged mechanical ventilation, age and low lymphocyte count	23
Kato et al	Japan	2011–2016	Retrospective, observational, multicenter study (5 hospitals)	Candidemia(all patients)	289	Follow-up blood culture,empiric treatment with fluconazole	Age > 65 years and SOFA score ≥ 6	5
Ala-Houhala et al	Finland	2007–2016	Retrospective, observational study (2 hospitals)	Candidemia(> 18 years patients)	374		Severity of underlying illnesses, ICU stay at the onset of candidemia and age > 65 years	4
Medeiros et al	Brazil	2011–2016	Retrospective, single-center, observational cohort study	Nosocomial candidemia	68		Older age, severe sepsis and hypotension	25
Santolaya et al	Chile	2013–2017	Prospective, observational multicenter, laboratory-based survey study(26 tertiary care hospitals)	Candidemia(all patients)	780		Mechanical ventilation and previous use of corticosteroids	17
Alkharashi et al	Saudi Arabia	2013–2018	Retrospective, observational, single-cente study	Candidemia(> 18 years patients)	324		Use of broad-spectrum antibiotics and use of central venous catheters	24
Xiao et al	China	2011–2017	Retrospective, observational, single-cente study	Candidemia(26–91 years patients)	82		GCS score, P/F ratio, MAP	36
This study	China	2013–2019	Retrospective, observational, single-cente, cohort study	Candidemia(0–5 years and > 16 years patients)	201	Length of hospital stay(days)	Respiratory dysfunction and Septic shock	This study

*CVC* central venous catheter; *APACHE* Acute Physiology and Chronic Health Evaluation; *ICU* Intensive care unit; *SOFA* Sequential Organ Failure Assessment; *GCS* Glasgow Coma Scale; *P/F ratio* PaO2/FiO2 ratio; *MAP* Mean arterial pressure

## Discussion

This was a 7-year retrospective study of candidaemia in a regional tertiary teaching hospital in Southwest China. We not only analysed the epidemiological characteristics, including the basic information of patients, underlying comorbidities, risk factors, the distributions of Candida species, antifungal agent use, antifungal agent susceptibility results and patient outcomes, but also performed epidemiological comparisons between paediatric patients and adult patients. To our knowledge, this is the first epidemiological comparative study of candidaemia between paediatric and adults patients in Southwest China, which provides reference data for the prevention and treatment of candidaemia in paediatric and adult patients.

Our data showed that there was no significant difference in the sex ratio, length of hospital stay or mortality between adult and paediatric patients (*P* > 0.05). However, the proportions of underlying comorbidities in paediatric patients, including pulmonary infection, neurological diseases, congenital malformations/syndromes and haematologic (nonmalignant) disease, were higher than those in adult patients (*P* < 0.05), and the other proportions in adult patients were similar or higher than those in paediatric patients (Table 2). Among the risk factors, only CVC, other invasive catheters and abdominal surgery in adult patients had higher risks than those in paediatric patients (*P* < 0.05), and other risk factors in children had higher or similar risks as those in adult patients (Table 2). Fewer univariate predictors of poor outcomes were identified for paediatric patients than for adults patients (4 vs 11 predictors), as shown in Table 4. This situation has not been clearly shown in other studies, and more epidemiological investigations are needed for confirmation. The incidence of candidaemia among paediatric patients was significantly higher than that among adults (*P* < 0.05) (Table 2); however, no significant difference in mortality was found between paediatric patients and adult patients (*P* > 0.05) (Table 2) in contrast to other studies [16, 17].

Our data showed that the median age of patients with candidaemia and the proportion of males were similar to those in other studies [8, 18–23]. Moreover, our study showed that the patients with candidaemia were hospitalised mostly in internal medicine wards, which is different from other studies that reporting hospitalisation mainly in ICU wards [8, 22, 24–27], but similar to other studies [28–31]. This phenomenon may be related to the demographic characteristics of the inpatients in our hospital, most of whom had more than two underlying diseases and were hospitalised in internal medicine wards. However, the incidence of candidaemia was still the highest in the ICU, similar to other studies [8, 30–34]. In accordance with other studies [17–19, 24, 25, 30, 32, 35, 36], *C. albicans* was the most common cause of candidaemia in the whole hospital, but the proportion of non-*C. albicans* infections was higher than that of *C. albicans* infections. Moreover, the proportions of *C. glabrata* in surgical, internal medicine and paediatric wards were the highest, which is different from other studies in China [18, 19, 35–37] but similar to other studies in other countries [4, 22, 27, 29, 32]. This may be due to the large number of elderly patients and the increasing use of azole antifungal agents.

Our data showed that the incidence of candidaemia increased from 0.20 episodes/1000 admissions in 2013 to 0.37 episodes in 2016 and then dropped to 0.26 between 2017 and 2019. The change in the annual incidence rate was mainly due to the change in the incidence rate in paediatric patients. The reasons may be due to the gradual easing of restrictions of China’s two-child policy since 2013. The number of geriatric pregnant women has increased annually, resulting in an increase in the incidence of neonatal diseases. The change trend was similar to that reported by Oeser et al. [38]. The overall morbidity and 30-day mortality in ICUs and hospitals in this study were similar to those in another hospital in this region of China [18], but lower than those in hospitals in other regions of China [35, 37] and other countries [5, 8, 16, 20, 21, 23, 25, 30]. The overall mortality rate of candidaemia has been reported to be 20–49% globally [39], and the mortality rate was 20.4% in our hospital, which is low compared to the global rate. This may be because the demographic characteristics and underlying diseases of patients in this region are different from those in other regions or countries, and few severe patients were admitted to our hospital.

With regard to resistance, resistance to FCA, ITR and VRC were common in *C. albicans* and non-*C. albicans* species (Table 3). In our study, AMB and 5-FC were highly active against all Candida species. In paediatric patients, the resistance rate of ITR was higher than that in adult patients, but the resistance rates of FCA and VRC were lower than those in adult patients; however, and the resistance rate of Candida species was no significant difference in satisfaction between paediatric and adult patients(*P* > 0.05). Moreover, FCA was highly active against all Candida species in paediatric patients and could be used in paediatric patients with candidaemia as a first-line agent. In the whole hospital, the resistance rate to azole was higher than those reported in other regions [18, 19, 36] and countries [17, 19, 25, 29, 30, 34]. This may be related to the long-term use of empirical prophylactic drugs by clinicians. Therefore, it was necessary to conduct an epidemiological analysis of antifungal agent susceptibility and guide clinicians to choose the rational antifungal agents to avoid the continuous increase in resistance rates.

In this study, septic shock was an independent predictor of 30-day mortality; which has been reported in many other studies [18, 35]. However, the other factors reported here have rarely been reported in other studies [35, 40–43], possibly because the demographic characteristics, underlying diseases and risk factors of the patients in our study were different from those in other studies; which may be the reason that the independent predictors and protective factors in this study were different from those in other studies [5, 35, 40–43]. The independent predictors and protective factors in different regions and countries are shown in Table 6.

The limitations of this study must be acknowledged. First, this was a single-centre retrospective study, and the total number of patients(166 adult and 35 paediatric patients) were small. Our data might be influenced by the number of patients, the level of medical intervention, and the distribution of patient types. Second, due to technical limitations of the clinical microbiology laboratory and the impact of hospital policies, no data on echinocandins were available in our hospital. Therefore, the results may not be generalizable to all patients with candidaemia in China.

## Conclusion

*C. albicans* was the main Candida species, but *C. glabrata* has become the second most common species in this region. FCA was the main antifungal agent for paediatric patients. AMB and 5-FC were highly active against all Candida species. The morbidity and mortality rates in elderly patients were the highest. Respiratory dysfunction and septic shock were independent predictors of 30-day mortality. Further multi-centre studies on candidaemia in different geographical regions in all patients should be conducted to help infection specialists assess the distribution and trends in patients with suspected fungal infections.

## Data Availability

The data set supporting the conclusions in this article is available from the corresponding author on reasonable request.

## Abbreviations

BSI: Bloodstream infection
ICU: Intensive care unit
PICU: Paediatric intensive care unit
NICU: Neonatal intensive care unit
USA: United States of America
ATCC: American type culture collection
MALDI-TOF MS: Matrix-assisted laser desorption/ionization-time of flight mass spectroscopy
FCA: Fluconazole
ITR: Itraconazole
AMB: Amphotericin B
VRC: Voriconazole
5-FC: Flucytosine
CVC: Central venous catheter
MIC: Minimal inhibitory concentration
OR: Odds ratio
CI: Confidence interval
